# The Position of St. Bartholomew's Hospital

**Published:** 1912-02-10

**Authors:** 


### THE POSITION OF ST. BARTHOLOMEW'S HOSPITAL.

As we pointed out so long ago as 1903, the course which those responsible for the administration of St. Bartholomew's Hospital at that date pursued by their policy of obstinate determination not to modify their plans, though they were riddled with criticisms resting on a solid basis, has reduced our oldest endowed hospital to the position of an ordinary mendicant in the hospital world. It was quite clear from the outset to men of business—and that is why the City of London has not given the steady and liberal support it would otherwise have extended to the great City hospital—that the obstinate refusal to take into consideration the position of the hospital as a whole in 1903, and to deal with it on business lines, must result within a few years in financial disaster. This forecast of City men is now abundantly justified by the reports of the Finance Committee and the able statement by the Treasurer on the financial position of this hospital, which was submitted to the General Court of Governors on February 1. Previous to 1908 a loan had been contracted with the bankers of £33,900. This sum was increased by further borrowings to meet deficiencies on income account in subsequent years, and the overdraft at the bank is now upwards of £40,000. A further sum of £10,000 will probably be needed from the bank to meet current liabilities before the end of the present year. This unfortunate financial position has arisen from several causes, chief of which has been the reduction of the hospital's income owing to the unbusinesslike policy pursued in 1902 and 1903, when the income was reduced by upwards of £9,000 a year owing to the realisation of investments, and borrowings and loans to provide the £255,000 sunk in the purchase of an additional site. The careful administration of the hospital's affairs previous to 1902 produced an excess of income over expenditure of about £2,000 a year, making the net deficit in revenue about £7,000 per annum. The Treasurer has, by re-letting, increased the rentals of the estate some £1,500 a year, but has had to borrow large sums to complete the new outpatient and pathological blocks, amounting to upwards of £42,000. The expansion of the work of the hospital, and the increased charges arising in consequence since 1905, represent an annual increased expenditure of some £5,000 a year. It would, therefore, appear that this hospital urgently needs an immediate increase in its revenue of some £10,000 a year, and a sum of £100,000 to liquidate its liabilities and free it from bank overdrafts and loans upon which heavy interest has to be paid at the present time.

Everybody must regret the circumstances which have brought about the present financial difficulties at St. Bartholomew's Hospital, and we make bold to say that now the present Treasurer has caused the appointment by the Governors of a Special Committee to advise the General Court of Governors as to the policy to be pursued to place this hospital once more upon a satisfactory basis, providing the report of this committee frankly and clearly states the facts and gives sureties that care will be taken to avoid any recurrence of the causes which have contributed to the present *impasse*, that the City of London will come forward, and the Governors of St. Bartholomew's Hospital also will come forward, and by collective action relieve this hospital from the lamentable financial difficulties which it might never have suffered had wisdom characterised its management during the early years of the past decade. We shall await with interest the report of the Special Committee, the members of which have placed upon them a very grave responsibility. That responsibility is no less than the continuance or removal of the present monetary difficulties at St Bartholomew's Hospital, for the ultimate issue must largely depend upon the wisdom and frankness with which they discharge the responsible duties entrusted to them by the Governors at this crisis.

